# Effects of Dexamethasone and Cox Inhibitors on Intracranial Pressure and Cerebral Perfusion in the Lipopolysaccharide Treated Rats with Hyperammonemia

**DOI:** 10.1371/journal.pone.0117416

**Published:** 2015-02-12

**Authors:** Johan Rohde, Hans R. Pedersen, Peter N. Bjerring, Fin Stolze Larsen

**Affiliations:** Department of Hepatology, A-2121 Rigshospitalet, University of Copenhagen, Blegdamsvej 9, 2100, Copenhagen, Denmark; Indiana School of Medicine, UNITED STATES

## Abstract

**Introduction:**

Systemic inflammation may affect the brain by aggravating the stage of encephalopathy and increasing intracranial pressure (ICP) especially if liver insufficiency with hyperammonemia is present. The aim of this study was to determine if the influence of concomitant hyperammonemia and lipopolysaccharide (LPS) on the brain can be prevented by dexamethasone and cyclooxygenase (COX) inhibitors.

**Method:**

Fifty-four male Wistar rats, 6 in each group, were divided into the following groups: Saline+saline; LPS (2mg/kg)+saline; LPS+indomethacin (10mg/kg); LPS+diclofenac (10mg/kg); LPS+dexamethasone (2mg/kg) in experiment A. Experiment-B included the following groups: LPS+NH3 (140μmol/kg/min)+saline; LPS+NH3+indomethacin; LPS+NH3+diclofenac and LPS+NH3+dexamethasone. ICP was monitored via a catheter placed in cisterna magna and changes in CBF were recorded by laser Doppler flowmetry.

**Results:**

LPS with and without NH3 induced a similar increase in plasma 6-keto-prostaglandin-F_1α_ (6-keto-PGF_1α_) concentration together with a concomitant rise in CBF and ICP. Indomethacin and diclofenac prevented the increase in ICP by LPS alone, and with the addition of NH3 the increase in both CBF and ICP, which was associated with a decrease in 6-keto-PGF_1α_. Dexamethasone only reduced the LPS induced increase in ICP but not CBF, and partly the 6-keto-PGF_1α_ plasma concentration in the combined setup.

**Conclusion:**

These data indicate that activation of cycloooxygenases is of central importance for development of cerebral hyperemia and high ICP during concomitant systemic inflammation and hyperammonemia.

## Introduction

Brain edema and intracranial hypertension frequently evolves in patients with acute liver failure (ALF)[1–3]. Besides the presence of hyperammonemia there is now accumulating evidence that pro-inflammatory cytokines are also of pathophysiological importance [4–9].

Pro-inflammatory mediators, such as the tumor necrosis factor alpha (TNF-α) and prostanoids (products of the cyclo-oxygenases (COX)), are upregulated in response to bacterial endotoxins (i.e. lipopolysaccharide (LPS)), decrease the vascular tone and causes peri-vascular edema in the brain [10–13]. In ALF patients severe infections is known to aggravate the stage of encephalopathy and increase intracranial pressure (ICP) [10]. The reason on how infection affects the brain in liver failure remains essentially unknown [4;14] but suggest that anti-inflammatory interventions could be of therapeutic value. In support of this view it has been shown that induction of mild hypothermia can prevent (and reverse) development of high cerebral blood flow (CBF) and ICP in ALF [3]. To further explore the effect of anti-inflammatory therapy we here used an established experimental model [15] to investigate if development of cerebral hyperemia and high ICP induced by co-administration of LPS and NH3. We here test if the cerebral hemodynamics can be prevented by the two COX inhibitors indomethacin and diclofenac, as well as by dexamethasone.

## Material and Methods

Male Wistar rats weighing 350–400g (Charles River, Sulzfeld, Germany) were used. Once received, they were housed in plastic cages, fed on a regular chow diet with free access to water. They were kept on a 12:12 light/dark cycle. The study was approved by the animal trial committee of the Danish Ministry of Justice.

### Experimental preparation

After a period of at least 5 days the animals were anesthetized with pentobarbital (50 mg/kg i.v.) and all efforts were made to minimize suffering. Catheters (PE-50) were placed in the femoral artery, femoral veins and in the peritoneum. Heparin (500 IU) was administered through the arterial catheter. The arterial catheter was connected to a pressure transducer which was reset at the mid-level of the body. The rats were tracheotomized and mechanically ventilated (Hallowell EMC; E-vet. Haderslev, Denmark) with a frequency of 60 per minute and a tidal volume of 5–10 ml. The respirator was connected to a capnograph allowing the expiratory CO_2_ level to be monitored. The temperature was monitored by an intra-abdominally placed thermometer and maintained at 37 ± 0.2°C by the aid of a heating blanket.

The rat was fixed in a stereo-tactic instrument; a scalp incision was made and two small boreholes were drilled. One borehole was used for the placement of a catheter (PE-10) in cisterna magna. The catheter was connected to a pressure transducer, which was reset at the mid-level of the body. The other borehole was used to place a laser Doppler probe (Probe 407; Perimed, Stockholm, Sweden) in the brain cortex for continuous measurement of blood velocity using laser Doppler flowmetry (LDF). Flowmetry was performed using a periflux laser Doppler System 5000 monitor (Perimed, Stockholm, Sweden). Continuous recordings of arterial blood pressure, ICP and LDF were stored in a computer using the software Perisoft (Perimed, Stockholm, Sweden). This software was used to calculate the relative change in LDF, which was used as an index of relative change in regional CBF, and to measure the average values of ICP and mean arterial blood pressure (MAP) at consecutive time intervals of 5 min. The cerebral perfusion pressure (CPP) was calculated according to the formula CPP = MAP—ICP.

### Experimental design

Experiment A

The effect of indomethacin, diclofenac and dexamethasone on LPS induced changes in CBF, ICP and CPP was evaluated in five groups each containing 6 rats.

1. Saline + (saline)* + saline
2. LPS + (saline) + saline
3. LPS + (saline) + indomethacin
4. LPS + (saline) + diclofenac
5. LPS + (saline) + dexamethasone

* The vehicle of ammonium acetate (NH3).

Experiment B

The effect of indomethacin, diclofenac and dexamethasone on LPS plus NH3 induced changes in CBF, ICP and CPP was evaluated in four groups each containing 6 rats.

1. LPS + NH3 + saline
2. LPS + NH3 + indomethacin
3. LPS + NH3 + diclofenac
4. LPS + NH3 + dexamethasone

### Experimental procedure

Before the experiment was started, a stable baseline of MAP, ICP and LDF was secured. Arterial blood levels of O_2_, CO_2_, pH, K^+^ and Na^+^ were measured by the use of a blood analyzer (ABL 505; Radiometer, Copenhagen, Denmark) before and every 30 min during the experiment. Once a stable baseline was obtained LPS (E. Coli 0127 B8, Sigma-Aldrich) dissolved in sterile saline (1 mg/ml) or vehicle alone was injected i.p. (1 mg/kg) and subsequently i.v. (1 mg/kg). Ammonium acetate (NH3) at a concentration of 140 μmol/kg/min or saline alone was then administered i.v. to the animals by an infusion rate of 2.3–2.6 ml/hour and the chronometer was started.

Indomethacin (Confortid; Activas, Copenhagen, Denmark) or diclofenac (Sigma-Aldrich) 10 mg/kg were administered i.v. 15 min after start, while dexamethasone (Fortecortin; Merck, Darmstadt, Deutschland) 2 mg/kg was administered i.v. 30 min before start. Sterile saline was used as vehicle in the control group.

The experiment was terminated after 70 min. Arterial blood was sampled and the animal was decapitated. Blood samples were centrifuged and the plasma were frozen in liquid nitrogen in heparin containing tubes.

### Measuring of plasma TNF-α

Plasma TNF-α levels were measured by an ELISA technique using a commercially available rat TNF-α immuno kit (Rat TNF-α BMS622; Bender MedSystems GmbH, Vienna, Austria). The enclosed protocols were used directly and all samples were run in duplicate.

### Measuring of plasma 6-keto PGF_1α_

6-keto prostaglandin F_1α_ (6-keto PGF_1α_), a stable metabolite of prostaglandin I_2_, was measured in the plasma using a commercially available EIA kit (6-keto PGF_1α_ EIA kit; Cayman Chemical Company, Ann Arbor, USA) and used as an index of COX activity. The enclosed protocols were used directly and all samples were run in triplet.

### Statistical analysis

One-way analysis of variance or Kruskal-Wallis one way analysis of variance on ranks with Tukey Test were used to compare between groups. One way repeated measures analysis of variance with Tukey Test was used to test within groups at different time intervals. Values are reported as mean ± SE. P<0.05 was considered to be significant.

## Results

Baseline values of ICP, MAP, and body weight were similar in all the studied groups of rats in the two experiments. The final PaCO_2_ values were within the normal range and differences between the groups were not observed. Although differences were observed between some of the groups, the final pH blood values were within the normal physiological limits in all groups (Table 1). The plasma sodium and potassium levels also remained within the normal range (Table 1).

**Table 1:** Baseline measurements and arterial blood gasses at 60 min.

		Baseline		60 min. Arterial blood			
Group	Weight (g)(g)	MAP (mmHg)	ICP (mmHg)	PaCO_2_ (mmHg)	pH	[Na+] (mM)	[K+] (mM)
S + S	379 ± 3	121 ± 4	2.6 ± 0.2	40 ± 1	7.45 ± 0.01	137 ± 1	4.2 ± 0.1
LPS + S	378 ± 14	120 ± 4	2.8 ± 0.4	39 ± 1	7.41 ± 0.01^a^	137 ± 1	3.9 ± 0.1
LPS + dexamethasone	375 ± 5	118 ± 4	2.6 ± 0.2	40 ± 1	7.46 ± 0.01^c^	139 ± 1	4.2 ± 0.1
LPS + indomethacin	368 ± 14	122 ± 4	2.9 ± 0.4	39 ± 1	7.46 ± 0.01^d^	138 ± 1	4.2 ± 0.1
LPS + diclofenac	386 ± 6	125 ± 6	2.9 ± 0.4	40 ± 1	7.45 ± 0.01	140 ± 1	3.9 ± 0.1
LPS + NH3 + S	380 ± 8	117 ± 6	2.9 ± 0.4	41 ± 1	7.39 ± 0.02^b^	135 ± 2	4.3 ± 0.1
LPS + NH3 + dexamethasone	386 ± 16	117 ± 5	3.3 ± 0.3	40 ± 1	7.40 ± 0.02	136 ± 1	4.6 ± 0.2
LPS + NH3 + indomethacin	379 ± 5	124 ± 1	3.0 ± 0.4	39 ± 1	7.46 ± 0.01^e^	137 ± 1	4.6 ± 0.1
LPS + NH3 + diclofenac	386 ± 12	120 ± 5	3.2 ± 0.4	39 ± 1	7.45 ± 0.01^f^	137 ± 1	4.6 ± 0.1

Note. Values are expressed as mean± SE. Mean arterial pressure (MAP), intracranial pressure (ICP).

^a,b^ P < 0.05 vs. S + S,

^c,d^ P < 0.05 vs. LPS + S,

^e,f^ P< 0.05 vs. LPS + NH3 + S

Saline (S), Lipopolysaccharide (LPS), ammonium acetate (NH3), saline (S), indomethacin (Indo), diclofenac (Diclo), dexamethasone (Dex).

### Effect of LPS on cerebral hemodynamics (ICP, CBF, CPP)

**Fig 1:**
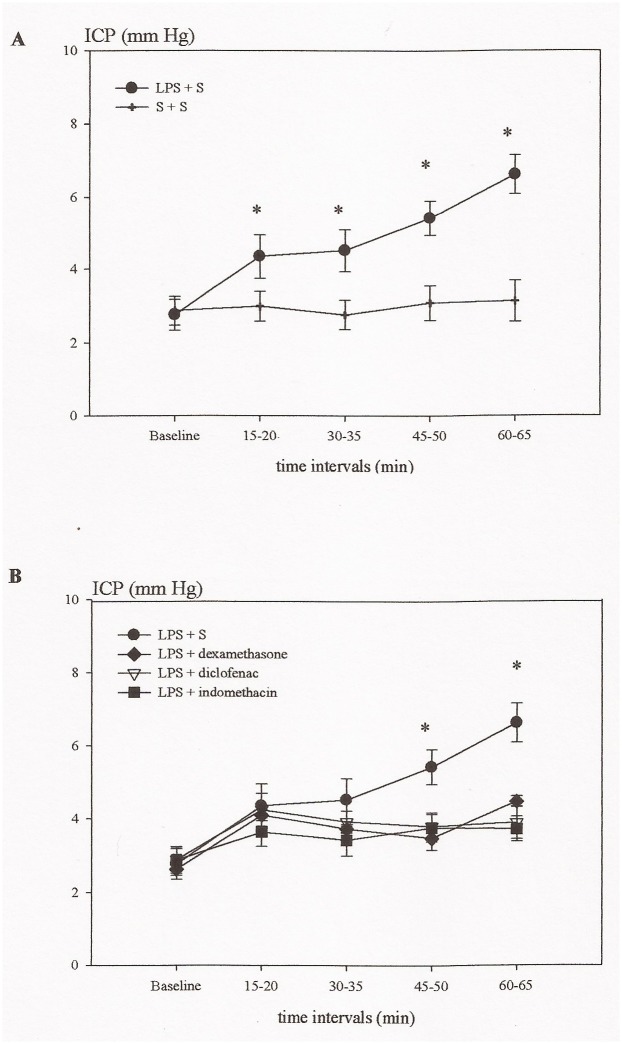
Time course showing the intracranial pressure (ICP) in the different groups of rats in experiment A. A: Lipopolysaccharide (LPS) induced an increase in ICP which in all the time intervals was significantly higher than the saline (S) + saline(S) group (*P < 0.05). B: Administration of indomethacin, diclofenac or dexamethasone significantly reduced the development of increased ICP induced by LPS (*P < 0.05).

**Fig 2:**
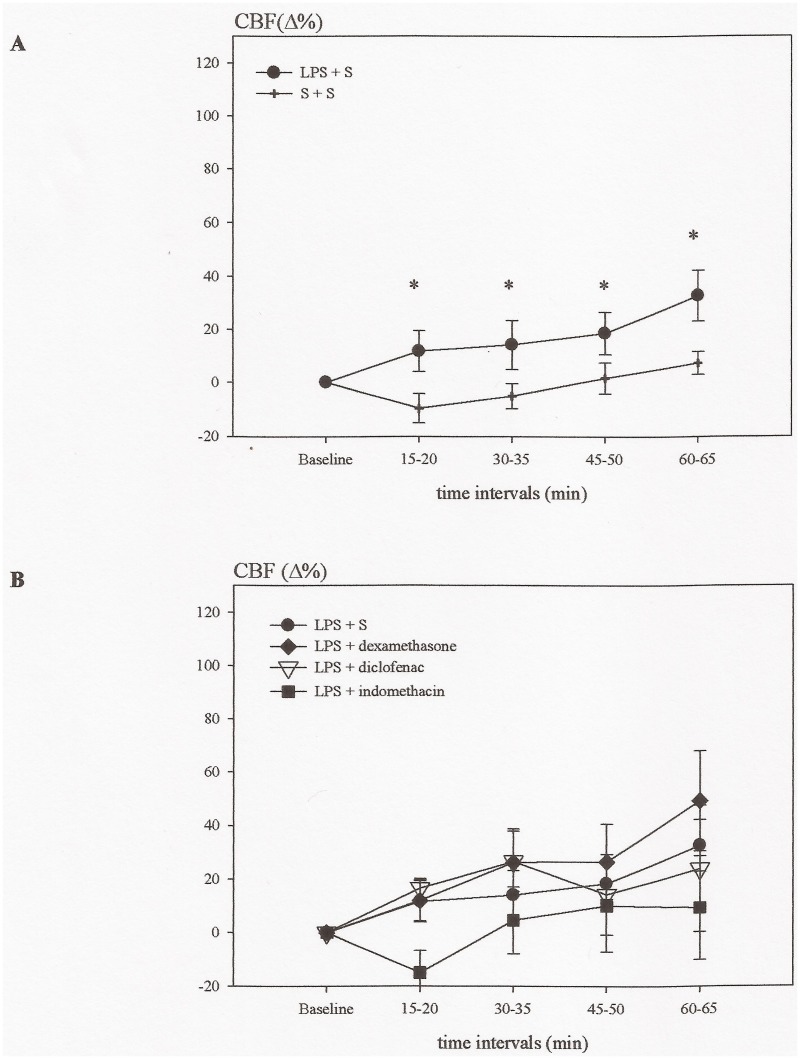
Time course showing the relative changes in cerebral blood flow (Δ%CBF) in experiment A. A: Lipopolysaccharide (LPS) induced an increase in Δ%CBF which was already present after 15 min. (*P < 0.05). B: Indomethacin, diclofenac and dexamethasone had no effect on the LPS induced changes of Δ%CBF.

**Fig 3:**
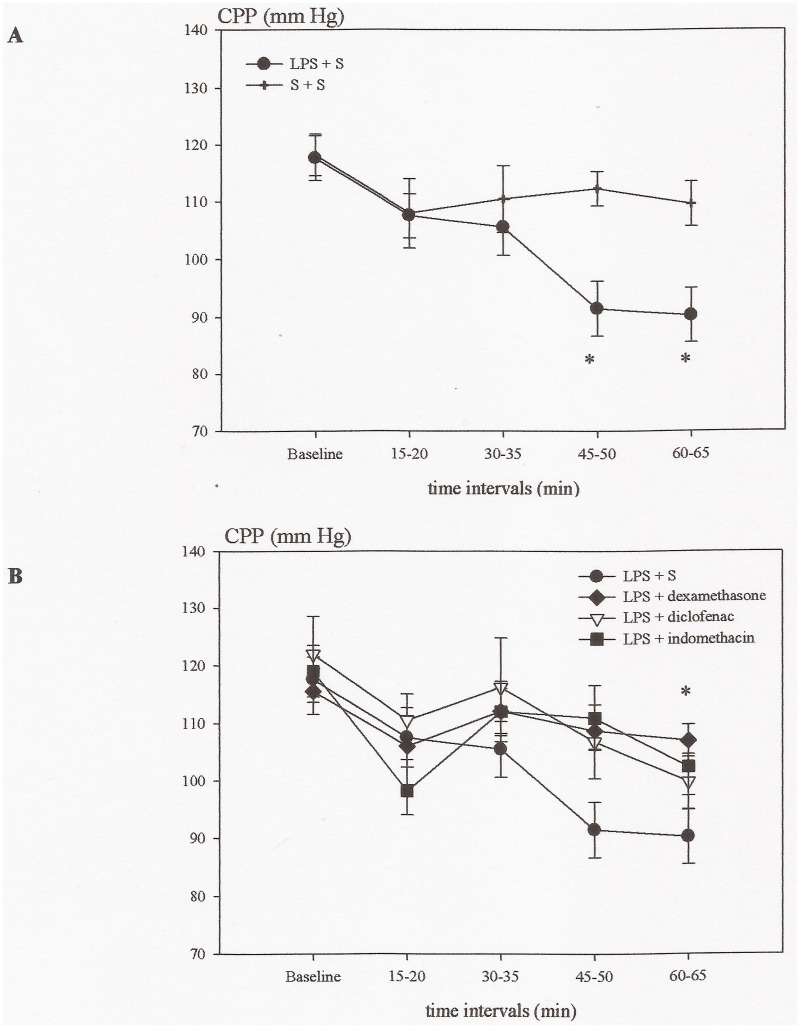
Time course illustrating the changes in cerebral perfusion pressure (CPP) in the different groups of experiment A. A: Compared to the baseline value CPP was lower in the two last time intervals in the Lipopolysaccharide (LPS) group (*P < 0.05), while no changes was observed in the saline (S) + vehicle (V) group. B. In the diclofenac administered group CPP also declined and was significantly lower at 60–65 min compared to the baseline value. Contrary, in the indomethacin, dexamethasone the CPP did not decrease.

Administration of LPS induced an increase in ICP (6.6 ± 0.5 mmHg in the final time interval) and CBF (33 ± 10% in the final time interval), which in all the time intervals was significantly higher than those of the control group (3.1 ± 0.5 mmHg and 7 ± 4% in the final time interval) (P<0.05) (Figs. 1 and 2). Contrary, the CPP gradually declined during the experiment (P<0.05) (Fig. 3).

### Effects of indomethacin, diclofenac and dexamethasone upon LPS induced changes in cerebral hemodynamics (ICP, CBF, CPP)

The two COX inhibitors indomethacin and diclofenac prevented the increase in ICP (3.7 ± 0.3 mmHg and 3.9 ± 0.5 mmHg after 60 min) (P<0.05), while they had no effect upon CBF (10 ± 20% and 24 ± 24% after 60 min) (Figs. 1 and 2). CPP decreased in the group treated with diclofenac at 60 min (P<0.05), while the decrease in the indomethacin group was not significant (Fig. 3). Dexamethasone significantly reduced the increase in ICP (4.5 ± 0.2 mmHg in the last time interval) (P<0.05), had no effect upon CBF (49 ± 19% after 60 min) (Figs. 1 and 2), but prevented the fall in CPP (P<0.05) (Fig. 3).

### Effect of indomethacin, diclofenac and dexamethasone upon LPS and NH3 induced changes in cerebral hemodynamics (ICP, CBF, CPP)

**Fig 4:**
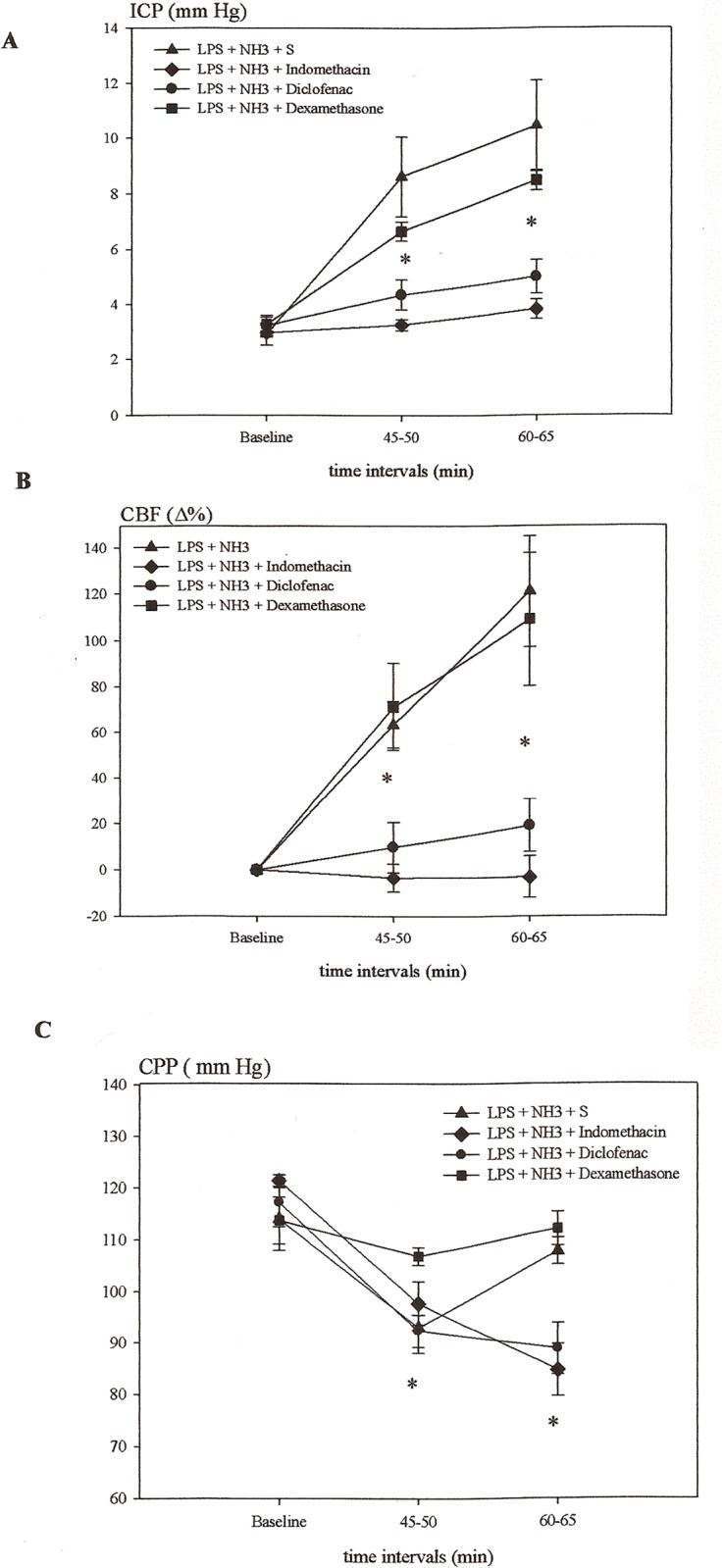
The effect of indomethacin, diclofenac and dexamethasone on changes of intracranial pressure (ICP), relative changes in cerebral blood flow (Δ%CBF) and cerebral perfusion pressure (CPP), and in rats exposed to lipopolysaccharide (LPS) and ammonium acetate (NH3). A + B: Indomethacin and diclofenac prevented the development of increased Δ%CBF and ICP (*P < 0.05) while dexamethasone did not have this effect. C: Compared to the baseline values CPP, in both time intervals, was reduced in the LPS + NH3 + indomethacin and LPS + NH3 + diclofenac groups (*P < 0.05), while in the NH3 + LPS group it returned to the baseline value in the last time interval.

The combined LPS and NH3 administration induced an increase in ICP (10.5 ± 1.7 mmHg after 60 min) and CBF (121 ± 24% after 60 min) which was substantially greater than that induced by LPS alone (P<0.01). This increase was prevented by indomethacin (3.8 ± 0.4 mmHg, -3 ± 9%) and diclofenac (5.0 ± 0.6 mmHg, 19 ± 11%) in the time intervals 45–50 min and 60–65 min (P<0.05) (Fig. 4). In the NH3 + LPS group CPP returned to the baseline value in the last time interval due to an increase in mean arterial pressure (data not shown). Compared to the baseline values CPP, in the time intervals 45–50 min and 60–65 min, was reduced in the LPS + NH3 + indomethacin and LPS + NH3 + diclofenac groups (P<0.05), while the administration of dexamethasone had no effect on changes in ICP and CBF but prevented the fall in CPP.

### Effect of indomethacin, diclofenac and dexamethasone on LPS and NH3 induced increase in plasma TNF-α and 6-keto PGF_1α_

Administration of dexamethasone ameliorated (P<0.05) the increase in plasma TNF-α while indomethacin and diclofenac did not have this effect (Table 2). The increase in plasma *6-keto PGF*_*1α*_ was completely prevented by indomethacin and diclofenac, while only partly by dexamethasone (Table 2).

**Table 2:** Plasma TNF-α and 6-keto PGF_1α_ at the end of experiment

Group	TNF-α (ng/ml)	6-keto PGF_1α_ (pg/ml)
S + S	0 ± 0	230 ± 20
LPS + S	56.10 ± 15.03^a^	3890 ± 600^a^
LPS + NH3 + S	66.57 ± 14.78^b^	3680 ± 220^b^
LPS + NH3 + Dexamethasone	25.84 ± 8.01^c^	1230 ± 170^c^
LPS + NH3 + Indomethacin	80.25 ± 9.43	90 ± 10^d^
LPS + NH3 + Diclofenac	61.15 ± 5.30	80 ± 10^e^

Note. Values are expressed as mean ± SE. Saline (S), Lipopolysaccharide (LPS), ammonium acetate (NH3).

^a,b^P<0.01 vs. S + S,

^c,d,e^P<0.01 vs. LPS + NH3.

## Discussion

The results of this study show that LPS induces a rise in both CBF and ICP, which are associated with a decrease in CPP, i.e. a decrease in the cerebrovascular resistance. An even more pronounced increase in both CBF (33 vs. 121%) and ICP (136 vs. 286%) was observed if also hyperammonemia was induced. These findings are in accordance with a previously conducted study using the same experimental model [15]. Also the effect of a superimposed inflammation induced by LPS on the brain in bile duct ligated rats shows similar results [5].

In this study we speculated that the increase in CBF and ICP induced by LPS with or without hyperammonemia could be explained by the influence of different pro-inflammatory mediators upon the brain vasculature causing modulation of the blood-brain barrier (BBB) with subsequently increased permeability and relaxation of the microvessel endothelial cells that control vascular tone [16]. In support of this view hyperemia has been reported to develop in naïve rats in response to iv infusion of TNF-α [14]. In the present study, the TNF-α plasma concentration increased following exposure to LPS which then make TNF-α a plausible candidate. This is in accordance with previously conducted studies showing that the plasma TNF-α concentration increases very fast following LPS injection [17]. Products of the COX enzymes, which are known to be activated in response to LPS, are also able to modulate the BBB microvessel endothelial cells and the smooth muscle cells of the brain arterioles [18]. The high plasma level of the stable prostaglandine metabolite 6-keto PGF_1α_ found in the present model confirms that the COX pathway is activated. Interestingly, the addition of ammonia, does not course any additional elevation in the TNF-α or 6-keto PGF_1α_ plasma concentrations indicating that the effect of hyperammonemia is not mediated through an elevation of the prostanoids or TNF-α concentrations.

The main finding in the present study was that the two unspecific COX inhibitors indomethacin and diclofenac prevent the increase in ICP in the LPS group, and the increase in CBF plus ICP in rats exposed to both LPS and ammonia. This neuroprotective effect of indomethacin and diclofenac in the LPS + NH3 group is associated with an almost complete prevention of plasma 6-keto PGF_1α_ formation, and seems in accordance with an experimental study that showed that indomethacin can prevent the development of hyperemia in portacaval administered rats exposed to NH3 infusion [19]. Contrary to the two COX inhibitors, dexamethasone did not prevent the increase of CBF and ICP induced by LPS and NH3. This finding may be explained by a weaker effect of dexamethasone in inhibiting the formation of vasoactive prostanoids as we found that the plasma level of 6-keto PGF_1α_ is only partly reduced compared to the LPS+NH3 group. We cannot refuse that administration of dexamethasone earlier than 30 minutes before the study was initiated might not have allowed the fully effect of dexamethasone. Further study will be needed.

Dexamethasone inhibits phospholipase A2 leading to a reduction in the free aracidonic acid, the substrate of the COX enzymes which explain an only partly reduction in 6-keto PGF_1α_ formation [20]. The observation, however, that the small but significant increase of CBF in the group treated with LPS alone is not reduced by the indomethacin and diclofenac, in contrary to ICP, may indicate that the mechanism underlying the development of high ICP and CBF are different when hyperammonemia is also present.

It could be questioned whether the effect of indomethacin is related to inhibition of COX or mediated by another mechanism, as hypercapnia-induced brain vasodilation can be prevented by indomethacin, while diclofenac fails to have same effect [21;22]. Obviously, the finding that diclofenac has the same effect as indomethacin in this study favors the involvement of vasoactive prostanoids in causing brain edema and high ICP since the PaCO2 levels in the study animals remained stable. Our results also indicate that the synergistic effect between ammonia and endotoxin on the cerebral hemodynamic depends on a high level of vasoactive prostanoids. However, the observation that the two COX inhibitors have no effect on the LPS induced increase in the TNF-α plasma level suggests that TNF-α is not a pro-inflammatory mediator that actually carries out, at least not alone, the changes in cerebral hemodynamics seen in the present model. A more likely role of TNF-α is that it works via a signal molecule placed longer downstream on the inflammatory cascade pathway, such as prostacycline [18;23]. Clearly more study is needed to firmly establish the key role of TNF-α in mediating cerebral vasodilatation and high ICP, since high levels of TNF-α play a major role in ALF gut-liver-brain axis and neurological complications [24;25].

In conclusion the results of the present study show that LPS (with and without hyperammonemia), induces an increase in the plasma concentration of both TNF-α and 6-keto PGF_1α_, which correlate with a rise in both CBF and ICP. The COX inhibitors indomethacin and diclofenac prevent the increase in 6-keto PGF_1α_ plasma concentration but not in TNF-α measured in the LPS + NH3 group. Furthermore, indomethacin and diclofenac, but not dexamethasone, prevents the synergistic effect of LPS and NH3 on CBF and ICP, which suggests a role of vasodilatory prostanoids in the development of cerebral hyperemia and high ICP in the naïve rat with concomitant systemic inflammation and hyperammonemia.

Taken together our data supports the thesis that anti-inflammatory intervention, that is COX inhibition, is a serious candidate for treatment of HE in ALF patients with hyperammonemia. One could speculate that treatment with COX inhibitors as well as dexamethasone would be beneficial. However, we couldn’t demonstrate any changes in the cerebral haemodynamics in the LPS + NH3 setup by dexamethasone and therefore we didn’t test the potential synergistic effect of NSAID and dexamethasone.

All physicians should however consider the potential detrimental impacts administrating steroids and COX inhibitors. It is widely known that prednisolone have a tendency to mask signs of infection, sometimes even reactivate infections [28], which is a major cause of morbidity and mortality in ALF patients. COX inhibitors contain several negative physiological effects in humans as well as in animals. For example they increase the risk of gastrointestinal haemorrhage by inhibiting the cytoprotective effect of prostaglandins (mucus production, bicarbonate secretion e.g.) thereby thinning the mucosal layer and increasing the risk of ulceration. Also, ALF patients often have thrombocytopenia and administration of COX inhibitors reduces platelet activation through decreased thromboxane A2 synthesis/activity inhibiting platelet aggregation resulting in impaired blood clotting ability.

Though sodium and potassium levels remained stable in our experimental model of ALF it is important to remember that cirrhotic patients with acute liver failure often suffer from impaired kidney function due to complex circulatory changes involving splanchnic vasodilatation, decreased effective circulatory volume, activation of the renin-angiotensin-aldosterone system and renal vasoconstriction (Hepatorenal Syndrome). By constricting the afferent arteriole in the glomerulus and reducing the glomerular filtration COX inhibitors could worsen kidney function even more rapidly.

Overall this study suggest that NSAIDs could be of importance for future treatment regimes in ALF by bringing the theory of the gut-liver-brain axis into account, in respect of bacterial translocation, gram negative bacteremia, liver macrophage activation and proinflammatory cytokines production [26,27]. We speculate that early anti-inflammatory intervention by COX inhibition could prevent the detrimental consequences of brain oedema and intracranial hypertension in ALF patients.
